# Effects of two years of physically active lessons on cognitive indicators in children

**DOI:** 10.1038/s41598-023-35644-0

**Published:** 2023-05-31

**Authors:** David N. Oliveira, Ellen Caroline M. Silva, Luciana L. S. Barboza, Mabliny Thuany, Raphael Henrique O. Araújo, Roberto Jerônimo S. Silva, Thayse Natacha Gomes, Heike Schmitz, Julian Tejada, Danilo R. Silva

**Affiliations:** 1 Federal University of Sergipe, São Cristóvão, Brazil; 2 State University of Londrina, Londrina, Brazil; 3 University of Brasília, Brasília, Brazil; 4 Faculty of Sports, University of Porto, Porto, Portugal; 5 Department of Physical Education and Sport Sciences, University of Limerick, Limerick, Ireland; 6 Physical Activity for Health Research Cluster, Health Research Institute, University of Limerick, Limerick, Ireland; 7 Faculty of Health Sciences, Universidad Autónoma de Chile, Santiago, Chile

**Keywords:** Physiology, Public health

## Abstract

Our purpose was to evaluate the effect of physically active lessons (PAL) on the cognitive performance of children during two years of follow-up. Four classes (second grade of elementary school) were divided into two intervention classes (n = 34) and two control classes (n = 27). Evaluations were performed before the intervention (M1), after 3 (M2) and 9 (M3) months in the 1st year, and 14 (M4) and 18 (M5) months in the 2nd year. The intervention was based on PAL integrated with the curricular components, which stimulated the children to stand or move in the classroom. Cognitive performance was evaluated using three computerized tests for response inhibition, selective attention, and cognitive flexibility. The children in the intervention classes presented improved cognitive performance in the execution of all tasks along the two years follow-up, in both correct answers and time reactions, with exception of correct answers of visual search. For the intervention classes, in most of the tasks, the mean differences confidence interval of 95% did not include the 0 on the two last moments of evaluation, and in all cases, the mean differences of them between M1 versus M5 were significantly different with high values of effect size (cohen -d > 1). PAL promotes modest improvements in diverse cognitive functions in children.

## Introduction

A high prevalence of sedentary behavior has been observed in the pediatric population, regardless of cultural aspects and the socioeconomic development of the country^1^. Studies have indicated harmful effects, and associations of long periods of sedentary behavior with the risk of developing future diseases^2^. In addition, this behavior seems to be negatively related to cognitive development during childhood, through prolonged alterations in brain structure and function^3–5^.

Considering the role played by the school in the full development of young people, including academic, affective-social, cognitive, and physical aspects, and that children remain in sedentary behavior for 64% of their time in this environment^6^, intervention studies have sought to increase levels of physical activity in this context^7^. Intervention strategies have sometimes been specific (such as in physical education, recess, or evening classes, for example) to compensate for this sedentary time^8^. More recently, interventions have sought to integrate physical activities with the content in classroom lessons. A systematic review noted that the majority of interventions with physical activity at school were focused on moderate and vigorous intensities and were shown to be efficient in raising children's cognitive performance, as well as reflecting on academic performance^9^.

However, when interventions with another typology are considered, such as interventions with physically active lessons (PAL) that incorporate physical activities into the pedagogical content of the classes, the cognitive effects are inconclusive^7^. PAL has been a viable option for implementing the movement in the school environment, specifically in the classroom, with good acceptance by students and teachers^10^. The majority of studies on PAL have been conducted in developed countries, with an educational and environmental structure favorable to the implementation of physical activities^7^. In these contexts, the teaching model is full-time schools, which means the time available for the development of these interventions is longer. When considering educational institutions from low-middle income countries such as Brazil, for example, the children are mostly at school for only one shift (four hours), or in the morning or the afternoon^11^. This hegemony of the educational context and type of intervention, limits the transfer of these results to other contexts, demonstrating the need for investigations to verify whether PAL are effective, and whether they can have positive effects on the cognitive functions of children from different school contexts^12^.

In addition to these aspects, a recent review showed that the average time of previous classroom interventions was up to six months^7^. Thus, it is possible that shorter periods may be insufficient to identify perceived effects on cognitive performance. Therefore, investigations are necessary with a longer follow-up period that investigates the effect of reduced sitting time in the school environment on children's cognitive performance indicators. Thus, considering the need to verify the effects of PAL on children's cognitive functions in educational contexts in low-middle income countries and over a longer period of monitoring, the objective of this study was to investigate the effects of two years of PAL on the cognitive indicators of children from Aracaju, northeastern Brazil. This study hypothesizes that PAL may promote improvements in children's cognitive performance over a two-year intervention.

## Methods

### Study design

**Figure 1:**
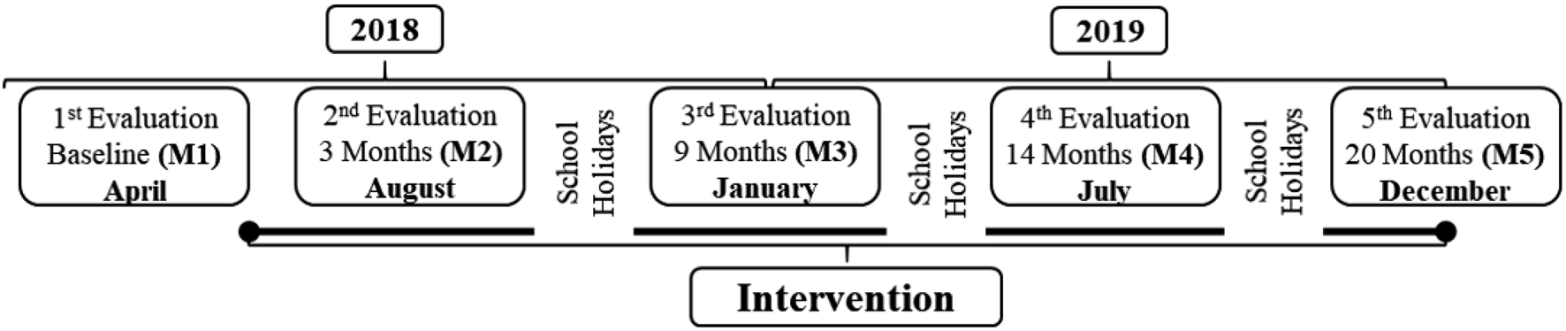
Timeline of the study.

This is a controlled trial with cluster sampling conducted in the school environment for two academic years. A school from Aracaju (Brazil) was chosen and all the second-grade of elementary school classes (n = 4) were included into the study. The four classes were divided into: intervention group (n = 34, two classes) and control group (n = 27, two classes). The school and classes were chosen by convenience. One evaluation was carried out before the intervention, two evaluations in the first year (2018), and two in the second year (2019). The interval between the first and second evaluations was 3 months, and between the first and third assessments, 9 months. In the second year, the interval between evaluations was 5 months (Fig. 1). The interval between the third and fourth evaluations, a 6-month period, occurred due to school holidays and the beginning of the 2019 school year, when the children advanced a grade. This study was described in accordance with the Consolidated Reporting and Testing Standards checklist for extension for randomized pilot and feasibility trials^13^.

### Sample

**Figure 2:**
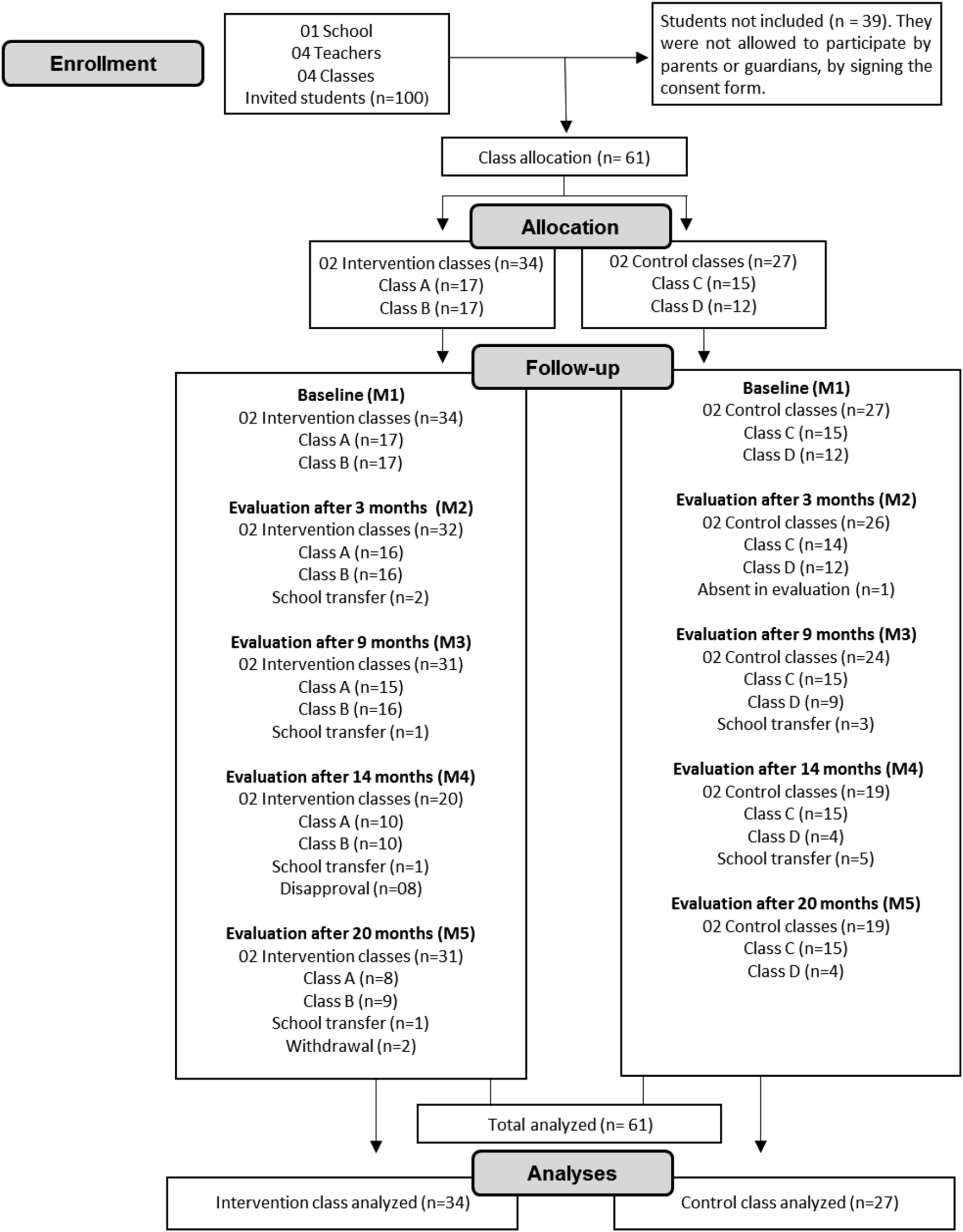
Flow of participants.

This study included 61 children of both sexes, with a mean age of 7.8 ± 0.5 years. All children regularly enrolled in the second grade of elementary school were invited. However, only those who presented authorization from parents or guardians to participate in the study, by signing the informed consent form, were included (Fig. 2). Children who had any severe mental disability or neurological disorder were not included in the study. This study was approved and followed all the guidelines of the Ethics Committee in Research with Human Beings of the Federal University of Sergipe (protocol nº: 2.587.676).

### Evaluations

Anthropometric measurements: Anthropometric measurements of body mass (kg) and height (m) were collected at the school, on a WISO portable scale and Sanny stadiometer, in an orthostatic position. The Body Mass Index (BMI) was calculated from the results of these measures.

Cognitive performance was evaluated using three distinct executive functions: inhibitory control, selective attention, and cognitive flexibility. Computerized tests were performed using the Psytoolkit platform^14,15^, version 3.2.0, and were supervised by a psychologist. The tests were appropriate to the age of the participants and the progression of difficulty was adjusted in the second year of intervention. The tests were performed in the morning and administered by psychology students trained to apply the tests. Children were taken individually from the classroom to the assessment room with the evaluator. The participants performed the tests in the order in which they are described, and for each test, two attempts were made consecutively, with the objective of familiarization, and for the evaluator to establish if the child had understood the test procedure. For the analysis of the results, the total number of correct answers and the time taken in the tests in milliseconds were considered as indicators. Only students included in the study sample were evaluated.

To evaluate inhibitory control, the computerized Go/NoGo traffic light test was used^16^. This test consists of the graphical presentation of several figures in the form of traffic lights, where the colors green and red are alternated at random. When the figure with the green traffic light appears, the child is required to press a computer key (Space). When the red traffic light appears, the child is required to wait for the figure to change, without pressing the key. The focus of this test is to evaluate inhibition of responses.

Selective attention was evaluated using the computerized Visual Search test^17^. This test graphically presents some figures with the letter T in various positions and randomly distributed. The participant is instructed to press a computer key whenever the letter T is in its normal position and in red, even if there is only one letter in this position and color, among many others. If the figure shows the letter T in a position other than the normal one or in another color (yellow), the participant is required to wait for the figure to change, without pressing the key. The purpose of this tool is to test reaction time, visual discrimination, inhibitory control, working memory, and object constancy.

Cognitive flexibility was evaluated using the computerized mental rotation test^18^. In this test the participant is required to choose the same figure as another presented to them from two options, however, in both options, the figures are rotated to the right or to the left. The participant is instructed to show the evaluator which figure is the same, even though it is rotated. This test is intended to evaluate spatial reasoning and visual discrimination. The time of execution of the mental rotation test was not considered, as the participants had difficulty handling the mouse to point to the figures.

### Intervention

The intervention lasted two academic years (2018–2019). In the first year, the intervention began immediately after the first assessment, which was at the beginning of the school year (May 2018). The intervention was interrupted during the school holidays in September, and resumed from October until January 2019, the end of the school year. In the second year, the intervention began in April 2019, and was interrupted during the school holidays in August, and resumed from September until December, the end of the school year. Activities with PAL were integrated into the curricular components of Portuguese, Mathematics, Geography, History, Sciences, and Arts, and encouraged children to at least stand, as well as moving around in the classroom while learning the contents.

In the first year of the intervention (2018), teachers from the respective classes selected for the study were invited to participate. After accepting, the teachers participated in weekly meetings with the research team at the local university. At these meetings, material was initially created with suggestions for dynamic activities, linked to the pedagogical content. This material, which was called as “activities chest”, was made available to intervention teachers at the school. Examples of activities for Math, Portuguese and Sciences lessons are described in Table 1.

**Table 1:** Examples of physically active lessons carried out by the intervention group.

PAL description	Expected movement
To exercise mathematical operations, several questions of arithmetic sums were written on the board, and students (in groups of five or six at a time) should write the answers on it	Stand up
	Arm movements
To exercise word formation, squares were drawn on the classroom floor, each with different syllables inside. Each student at a time should move, jumping inside the squares and choosing syllables to form words	Stand up
	Displacements with jumps
To exercise selective collection for recycling, students were divided into groups and stood around a table. They should paste on cards shaped like garbage cans, with colors red, yellow, green, and blue, the figures with objects that symbolized glass, plastic, paper, and tin, in the colors corresponding to the proper disposal	Stand up
	Arm movements
To exercise the punctuation marks, the students received a sheet indicating combinations of movements for each punctuation mark. They should elaborate sentences and after reading, finish with a movement that corresponds to the correct punctuation. For example, a question mark would be represented by leaning the torso forward and downwards, a point indicated by squatting and stretching with an exclamation	Stand up
	Arms, legs, and torso movements

The meetings with these teachers took place throughout the academic year, where doubts were clarified, in addition to discussing aspects related to the implementation of the intervention.

In the second year of the intervention (2019), with the transition of the children to the third grade, there was a change of teachers. The new teachers of the intervention classes were invited to insert the activities in their lesson plan and were also invited to weekly meetings with the research team. In order to receive assistance and answer questions about the implementation of activities, a team member also accompanied them weekly during their empty time window because the pupils had physical education classes taught by another teacher. They also received a specific “activities chest”, prepared by the research team based on the textbook of the disciplines. All teachers (2018 and 2019) were instructed to include the activities from the chest as many times as possible, with a goal of at least 15 min/day over 3 days/week. Students who belonged to the intervention classes, but were not included in the study, also participated in the activities with PAL.

This study was described in accordance with the Template for Intervention Description and Replication (TIDieR) reporting guidelines and additional details of the intervention are presented elsewhere^19,20^.

The control group was advised to maintain the traditional teaching model following the municipality-recommended guide, participating only in the assessments.

### Statistical analysis

The data were treated in SPSS 25.0 (IBM Corp. Armonk, NY, USA), and descriptive analyses (mean, standard deviation, absolute and relative frequencies) were performed to characterize the sample and to describe the variables in the initial evaluation. The normality of the data was evaluated using the Kolmogorov Smirnov test, comparing the measures between the control and intervention groups in the initial assessment. The t-test was performed for independent samples or the Mann–Whitney U test, according to the data distribution. For comparison of categorical data (sex), the chi-square test was performed. The measures of the cognitive tests were analyzed through the number of correct answers and the time of execution (milliseconds), with the exception of the time in the mental rotation test.

Initially, 61 children participated in the study, but only 36 (intervention n = 17, control n = 19) completed all tests at all evaluation moments. Children who had data from at least one evaluation moment were included in the analyses. To compare the five evaluation moments between the two groups (intervention and control), crude and adjusted models of Generalized Estimation Equations were performed, followed by the Bonferroni post hoc, and adopting 95% of confidence interval. For the adjusted models, the age and values of the initial assessment (baseline) were tested as covariates, however, only those variables that were significant for the model were included.

The effect size was calculated from Cramer's V equation. The calculation of the size of Cramer's V effect was done through the square root of Wald chi-quadrat divided by the total sample number (n = 61) multiplied by the value of the degree of freedom (df) found in the group and time interaction. Therefore, small effect values up to 0.05, mean values above 0.05–0.15, and large values of 0.25 were considered.

Additionally, tests using the estimation approach Calin-Jageman & Cumming^21^ were performed to compare the results of all cognitive tests along the moments, using a bootstrapping model of 5000 re-samples with replacement and estimating *p*-values and Cohen-d for all comparisons. Those analyses were performed using the Dabest package for Python.

## Results

Table 2 presents the sample characterization at baseline. Differences between groups were observed for the variables age (*p* = 0.01) and execution time of the Go/NoGo test (*p* = 0.04).

**Table 2:** Characterization of the sample at baseline.

Variables	Intervention (n = 34) Mean (SD)	Control (n = 27) Mean (SD)	*p*
Female (%)	13 (38.2%)	14(51.9%)	0.42
Male (%)	21 (61.8%)	13 (48.1%)	
Age (years)	7.7 (0.5)	8.0 (0.7)	**0.01**
Weight (kg)	25.3 (3.9)	27.6 (6.2)	0.10
Height (cm)	120 (0.04)	130 (0.1)	0.38
Hits Go/NoGo	23.6 (1.5)	24.4 (0.9)	0.16
Time Go/NoGo (ms)	869.9 (199.7)	756.5 (222.1)	0.04
Hits visual search	7.1 (1.7)	7.8 (1.9)	0.16
Time visual search (ms)	3489.4 (1113.1)	3172.2 (935.0)	0.24
Hits mental rotation	12.1 (1.4)	12.9 (1.6)	0.07

Values described as mean and SD (standard deviation), except for sex (absolute and relative frequency).

**Figure 3:**
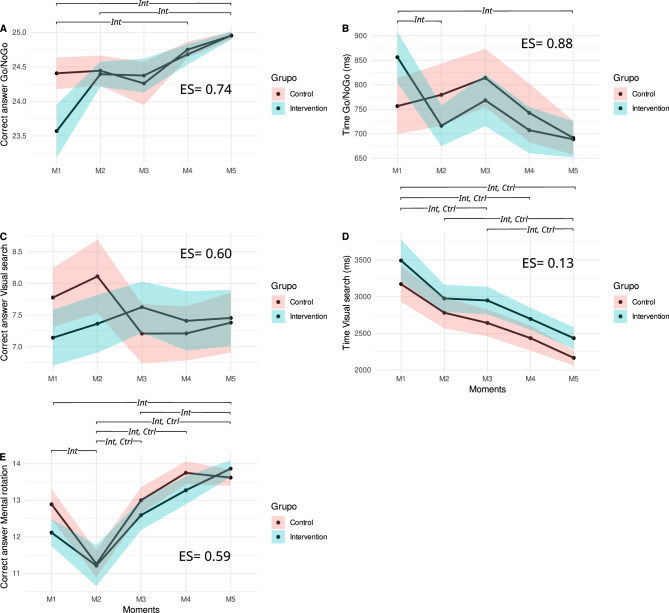
Comparison between evaluation moments and groups, in the indicators of children's cognitive function over two years of follow-up of an intervention with physically active lessons (n = 61). (Note: ES = effect size. Int = significant differences in the intervention group in the indicated moments. Ctrl = significant differences in the control group in the indicated moments).

Figure 3 presents the differences in the means between the variables analyzed at the five evaluation moments (M1, M2, M3, M4, and M5) according to the groups (control and intervention). Supplementary Figs. 1, 2, and 3 and supplementary Tables 1, 2, 3, 4, and 5 present the results of the interval estimation technique comparing the baseline versus the other moment evaluations of control and intervention groups. In general, both groups showed an improvement in their task performance in terms of frequency of correct answers and time reactions along the evaluations. However, the performance of the IG was better when compared to the evolution of their execution along the evaluation moments.

In general, the group factor was significant concerning the number of correct answers (*p* = 0.007), the time of execution of the Go/NoGo test (*p* = 0.010) and the time of execution of the Visual search test (*p* = 0.040), showing that the classes were different considering the two years of monitoring. In the time factor, the results were significant in the Visual search test (execution time, *p* < 0.001) and the number of correct answers in the Mental rotation test (time, *p* < 0.001), indicating that the groups improved over time considering all the moments of evaluation.

Regarding intragroup differences, in the Go/NoGo cognitive test, only the intervention group had a greater number of correct answers, at two moments, M4 and M5, (*p* < 0.001 for both moments), in relation to baseline and a higher number of correct answers at M5 when compared to M2 (*p* = 0.005). In the execution time of the Go/NoGo test, the intervention group improved significantly at M2 and M5 when compared to the baseline.

Both groups presented improved cognitive performance in the execution time of the Visual search test at M3, M4 and M5 (intervention, *p* = 0.015; *p* < 0.001; *p* < 0.001 and control, *p* = 0.012; *p* < 0.001; *p* < 0.001, respectively). The intervention group performed the Go/NoGo test in a shorter time compared to the control group (group vs time interaction *p* = 0.019), specifically in the second (M2) and fifth (M5) evaluation moments (see Supplementary Table 1). The groups remained statistically equal at all moments for the other variables, presenting non-significant variations in cognitive performance (Go/NoGo [hits] *p* = 0.078; Visual search [hits] *p* = 0.237; Visual search [execution time] *p* = 0.992; Mental rotation [correct] *p* = 0.249).

In the cognitive mental rotation test, the intervention group presented significant improvements at M5 (*p* < 0.001) and the control group at M2 (*p* < 0.001) compared to the baseline. Both groups presented significant improvements at M3, M4, and M5 when compared to M2 (intervention, *p* = 0.004; *p* < 0.001, *p* < 0.001, respectively, and control, *p* < 0.001 for the three moments). Only the intervention group improved significantly at M5 when compared to M3 (*p* = 0.014).

Regarding the Go/NoGo test, the frequency of correct answers and the time reaction of the intervention group showed a pattern of improved performance from the M2 with confident intervals of the means differences between baseline and the other moments that do not include the 0 (see Supplementary Fig. 1A and C), with significant *p*-values and high cohen-d effect size values (see supplementary Tables 2 and 3) when compared with the performance of the control group, which only showed a similar pattern (CI and *p*-value) for the M5. The only exception was observed in the M2 time reaction in which the CI of the difference between M2 and M1 for the intervention group include the 0 and the *p*-value was greater than 0.05, however, even in this case the differences between M2 and M1 showed a trend in which as time went by, the intervention group participants performance improved, a pattern that did not found in the control group.

The visual search test showed a similar pattern of improved performance regarding the time reactions for both intervention and control groups. Their CI (which does not include the 0), *p*-values and cohen-d (see Supplementary Fig. 2C and D and supplementary Table 5) demonstrate significant differences from M2 for IG and from M3 for CG until the last evaluation. However, no significant differences concerning the frequency of correct answers either for the intervention or control group were found for the visual search (see Supplementary Fig. 2A and C and supplementary Table 4).

The mental rotation test also showed a similar pattern to the Go/NoGo test, but only for the M4 and M5 evaluations in the intervention group with CI and *p*-values compatible with significant differences (see Supplementary Fig. 3 and supplementary Table 6), a pattern that was not found in the control group, which only presented significant differences for the M4 versus M1 comparisons (see Supplementary Fig. 3A and C and supplementary Table 6).

We identified a large effect size for the variables of Correct answer Go/NoGo 0.74, Go/NoGo Time 0.88, Correct answer Visual search 0.60 and Correct answer Mental rotation 0.59, except for the visual search test execution time variable, which presented a small value of 0.13 (Supplementary Table 7).

## Discussion

The main result of the study was the large effect size that we identified in almost all tests, with the exception of the visual search test runtime. The cognitive function of the students who participated in the PAL increased, with significant improvement in correct answers and time reaction in the three cognitive tests used. The only exception was the frequency of correct answers for the visual search, a highly difficult test for which the participants showed improvement in reaction time but not in the correct answers. In all other cases, it was possible to find meaningful differences in the expected directions: baseline with the lower average number of correct answers and higher average time reactions than at other moments, showing a gradual improvement over time.

This improvement was higher in IG than CG (lowers *p*-values and higher cohen-d values) when compared the differences between baseline and all other moments. The majority of previous studies that associate physical activity and cognitive function were based on the possible isolated effects of physical activity practice, especially of moderate and vigorous intensity^5,22^. The most consistent evidence has shown that most interventions focus on increasing physical activity at these intensities (moderate and vigorous) outside the classroom, indicating positive outcomes related to cognitive performance^12^. However, these positive results were generally found in developed countries, with full-time education and favorable socioeconomic conditions^9^. In contrast, our findings showed positive effects of PAL in one of the five cognitive indicators evaluated.

Inhibitory control is an important cognitive function for the development and academic performance of children. Previous studies have found positive associations between academic performance and cognitive performance in tests that evaluate children's inhibitory control^23^. Suggest that inhibition enables proficient performance of other executive functions, which, in turn, influences the ability to produce behaviors aimed at new goals, in new situations. In this way, it is possible that the stimuli of physical activities in classes can promote effects on inhibitory control in children, by allocating neural resources of attention through an increase in the plasticity of the frontal regions of the brain^24^.

In this perspective, school plays a fundamental role in the integral development of students, demonstrating that this environment could be a possible enhancer of cognitive performance, even in less developed countries^19,20^. Furthermore, the level of evidence on the likely advantages of replacing sitting time with light physical activity integrated with the curriculum content on cognitive functions remains low or inconclusive^8^.

It is important to note that, besides the similarity of effects in most cognitive variables compared with the control group, the children's performance of the intervention group was not inferior in any variable during the two years of follow-up. This means, on the one hand, the intervention did not harm students’ learning. On the other hand, the more PAL which also include more social interaction between students can, on long terms, contribute increasing their eagerness to learn. This is another aspect to be also investigated henceforth.

Considering the natural development of children, it is expected that cognitive performance will change based on the interaction with a set of factors, including the learning process in the school environment and other experiences in the family and social environment^25^. For this reason, the cognitive tests were adjusted in the second year of intervention (M4 and M5), making them more challenging, to minimize the possibility of a positive effect caused by children's predictable cognitive development^14,15^.

The recent review by Norris^7^ showed that the PAL observed with an average duration of 26 min per class, three to five days a week, and an average intervention time of six months did not indicate any noticeable effects on cognitive functions. Similarly, Watson^22^ found inconclusive effects on cognitive functions but noted that a possible explanation may be related to the variation in the quality of the tests used. The tests used in the present study are well-known and widely used and all measures in our study were administered by specialized professionals in the field of psychology, through computerized cognitive tests, adjusted for children of this age group. However, in this type of intervention, the teacher plays a fundamental role, because they are the ones who will impart the PAL in the classroom, and this is not common in the traditional culture of the school, where movement is generally not integrated into the curriculum components^26–28^. In this sense, it is essential to consider the involvement of teachers for better outcomes^29^.

In the current study, teachers were instructed to replace conventional lessons as often as possible, autonomously. Unlike other studies, in which teachers are directed to insert a rigid number of PAL, in the current study, teachers were free to insert the activities whenever they wanted, to preserve the external validity of this type of intervention. Despite this free choice, teachers reported applying the PAL three times a week, with an average time of approximately 45 min. However, because there was no daily control of how many times the activities were inserted, it is possible that the conduction of activities may have occurred at different frequencies and periods of time throughout the school year, in special due to the change of teachers after the grade transition. In this sense, interventions with PAL should be better reported in future studies, with detailed descriptions of how teachers carry out PAL, as well as the evaluation of cognitive indicators^7^.

The variability in the applied cognitive tests is a factor to be considered, since different measures can show different outcomes^30^. However, this condition may be a strong point of our study, as validated tests were used, and administered by trained evaluators. Evidence indicates that computerized tests provide greater consistency in administration and scoring, as well as greater precision in stimulus control and greater precision in measurement^30^. In addition, our main analysis eliminates the impact of sample loss, ensuring the quality of our results.

The findings of this study provide important practical implications for teachers, schools, and educational sectors. Since we observed modest but promising effects of PAL on cognitive function, the adoption of this new strategy to integrate movement into the school curriculum may assist the children’s academic goals, but also their full development through more attractive lessons and a less sedentary day. It is important to highlight the need of training with teachers considering the different educational contexts and the social support (e.g., educational sectors, schools principals, families) to implement this new paradigm at scale. In addition, gradual procedures could help a successful implementation of PAL in the medium- and long-term.

The limitations of the current study include: (a) the lack of information regarding behaviors performed outside the school environment during the intervention period; b) other domains of cognitive functions should also be explored; (c) the convenience strategy to select the sample should be considered for the results extrapolation; (d) the small sample size limit stratified analysis, such as by sex or classes; (e) although we have adopted a comparison group, the individual development effect could be impacted the cognitive performance over time; and (f) as teachers had the autonomy to implement the PAL and the study was conducted over two school years, the intervention might have had different according to the teachers' characteristics and it was intermittent over the follow-up period. Therefore, studies are needed to qualitatively investigate the involvement of teachers in the intervention, and how the PAL are carried out, using a tool that makes it possible to observe the number of lessons administered per class in its effects on cognitive outcomes.

## Conclusion

Interventions with physically active lessons seem to promote modest improvements in the cognitive functions of some children. However, the incorporation of pedagogical elements to make classes more physically active is a major challenge, which requires the coordination and training of teachers. The cognitive effects of these interventions are not easy to detect, and evaluation of other cognitive dimensions may be necessary to detect their possible effects. The findings of the current study also draw attention to the need for future studies to monitor classroom activities, with control of confounding variables in order to identify sustainable and effective strategies in the school context.

## Supplementary Information

Supplementary Information.

## Data Availability

The datasets used and/or analyzed during the current study are available from the corresponding author on reasonable request.
