# Supplementary material for: Effects of two years of physically active lessons on cognitive indicators in children
Source: Sci Rep. 2023 May 31;13:8774. doi: 10.1038/s41598-023-35644-0 (PMC10232448; doi:10.1038/s41598-023-35644-0)
Supplement: Supplementary file 1 — Supplementary Information. [file 41598_2023_35644_MOESM1_ESM.docx]

Supplementary materials

Calin-Jageman & Cumming estimation approach to compare the results of all cognitive tests across the time points represented in figures 1, 2 and 3, using a bootstrap model of 5000 resamples with substitution and estimating p-values and Cohen-d for all comparisons.


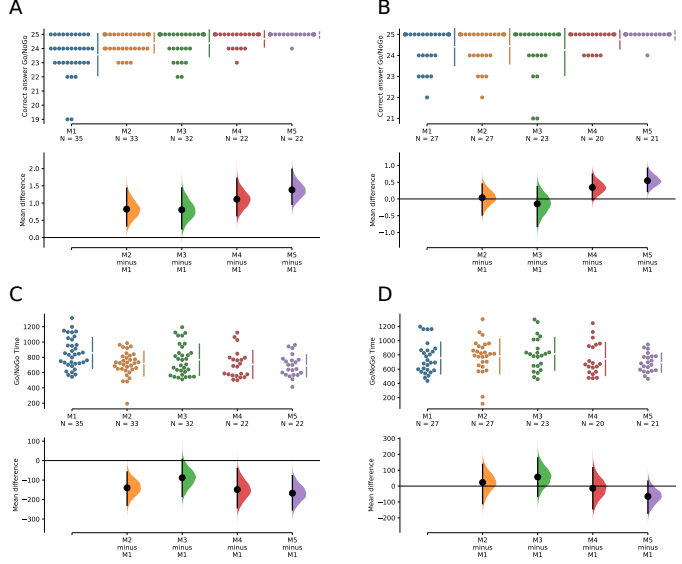


**Supplementary figure 1**. Confidence interval, p-value, and Cohen's d-estimate plots for mean differences in correct answers and Go/NoGo test reaction time between baseline and all time points. Blue Color: M1. Color Orange: M2. Green Color: M3. Red color: M4. Color Purple: M5.


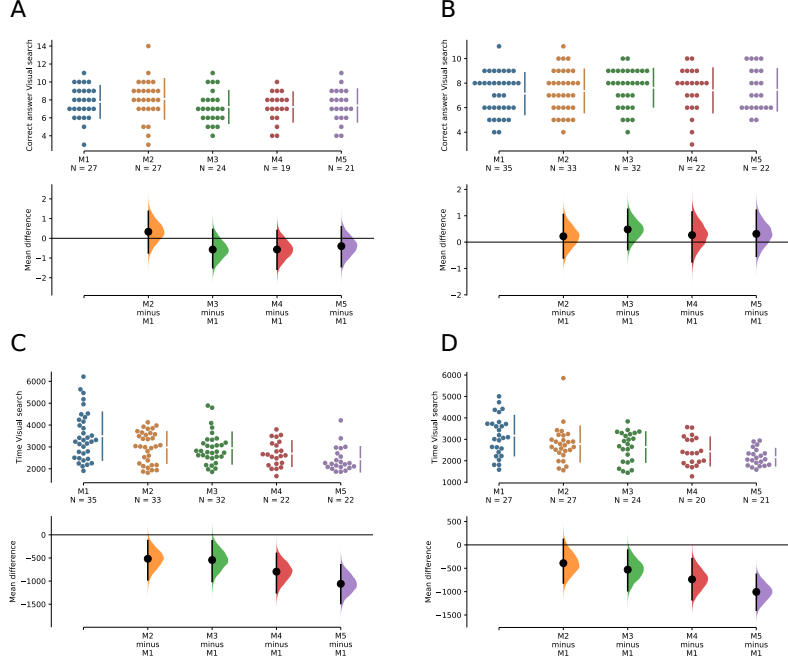


**Supplementary figure 2**. Confidence interval, p-value, and Cohen's d-estimate plots for mean differences in correct answers and visual search test reaction time between baseline and all time points. Blue Color: M1. Color Orange: M2. Green Color: M3. Red color: M4. Color Purple: M5.


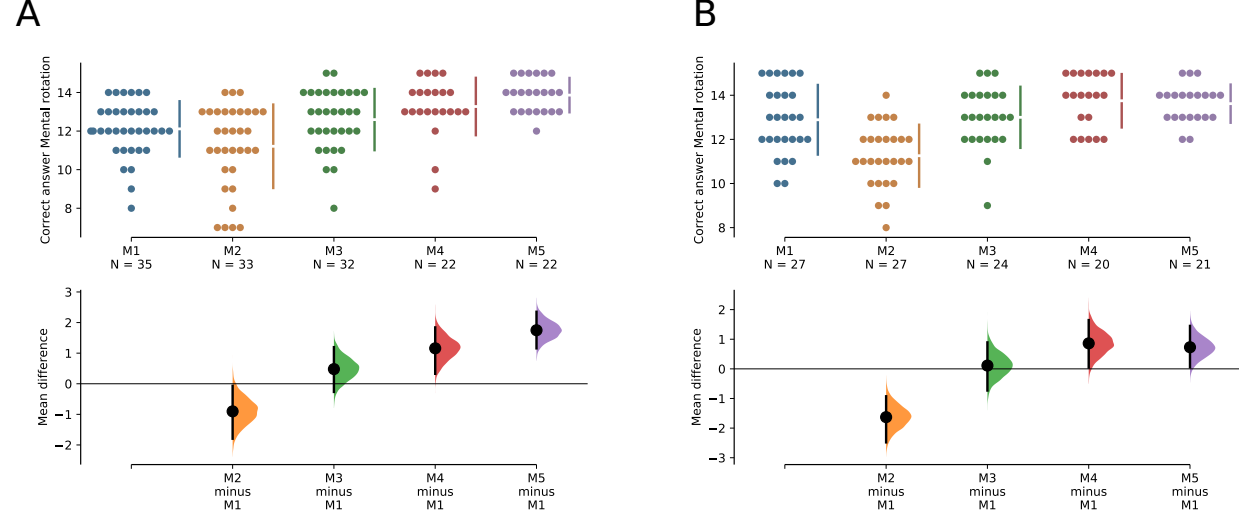


**Supplementary figure 3**. Confidence interval, p-value, and Cohen's d-estimate plots for mean differences in correct answers and mental rotation test reaction time between baseline and all time points. Blue Color: M1. Color Orange: M2. Green Color: M3. Red color: M4. Color Purple: M5.

**Supplementary table 1.** Comparison between variables and evaluation moments.

|  | Intervention | Control | p | | |
| --- | --- | --- | --- | --- | --- |
|  |  |  | Group | Time | Interaction |
| CA. Go/NoGo^In^ |  |  |  |  |  |
| M2 | 0,8  (-0,01 a 1,5) | 0,04  (-0,7 a 0,8) | **0,007** | **<0,001** | 0,078 |
| M3 | 0,8  (-0,08 a 1,7) | -0,2  (-1,1 a 0,8) |  |  |  |
| M4 | **1,03***  **(0,3 a 1,8)** | 0,3  (-0,2 a 0,9) |  |  |  |
| M5 | **1,3*^a^**  **(0,5 a 2,1)** | 0,5  (-0,03 a 1,0) |  |  |  |
| T. Go/NoGo (ms)^In^ |  |  |  |  |  |
| M2 | **-154,7***  **(-267,0 a -42,5)** | 23,1  (-133,4 a 179,6) | **0,010** | **<0,001** | **0,019** |
| M3 | -97,5  (-275,2 a 80,1) | 69,7  (-87,3 a 226,8) |  |  |  |
| M4 | -160,3  (-336,2 a 15,6) | 12,1  (-167,6 a 191,8) |  |  |  |
| M5 | **-166,4***  **(-300,8 a -32,1)** | -43,8  (-165,4 a 77,8) |  |  |  |
| CA. Visual search^In^ |  |  |  |  |  |
| M2 | 0,1  (-1,3 a 1,5) | 0,3  (-1,5 a 2,1) | 0,714 | 0,855 | 0,237 |
| M3 | 0,5  (-0,8 a 1,9) | -0,6  (-2,5 a 1,3) |  |  |  |
| M4 | 0,3  (-1,2 a 1,8) | -0,6  (-2,3 a 1,1) |  |  |  |
| M5 | 0,3  (-1,0 a 1,6) | -0,4  (-2,1 a 1,2) |  |  |  |
| T. Visual search (ms)^In^ |  |  |  |  |  |
| M2 | -512,3  (-2,5 a 1027,1) | -390,5  (-163,0 a 944,1) | **0,040** | **<0,001** | 0,992 |
| M3 | **-554,8***  **(-65,4 a 1043,9)** | **-530,2***  **(72,3 a 987,9)** |  |  |  |
| M4 | **-792,3***  **(307,7 a 1276,7)** | **-737,2***  **(233,4 a 1240,8)** |  |  |  |
| M5 | **-1054,3*^ab^**  **(463,4 a 1645,0)** | **-1006,3*^ab^**  **(470,4 a 1542,1)** |  |  |  |
| CA. Mental rotation^In^ |  |  |  |  |  |
| M2 | -0,7  (-2,1 a 0,6) | **-1,6***  **(-2,5 a -0,7)** | 0,531 | **<0,001** | 0,249 |
| M3 | 0,6^a^  (-0,6 a 1,7) | 0,05^a^  (-1,0 a 1,1) |  |  |  |
| M4 | 1,0^a^  (-0,1 a 2,1) | 0,8^a^  (-0,2 a 1,7) |  |  |  |
| M5 | **1,6*^a^**  **(0,6 a 2,5)** | 0,6^a^  (-0,6 a 1,9) |  |  |  |

**Supplementary table 2.** Confident interval, p-value and cohen d estimated for the mean differences between the baseline (M1) and the rest of the moments estimated using a bootstrapping procedure of 5000 re-sampling with replacement for the correct answers in the Go/NoGo test.

|  | Intervention group | | | Control group | | |
| --- | --- | --- | --- | --- | --- | --- |
|  | 95%CI | p-value | cohen-d | 95%CI | p-value | cohen-d |
| M2 - M1 | 0.333, 1.43 | 0.0042 | 0.703 | -0.481, 0.444 | 0.744 | 0.0427 |
| M3 - M1 | 0.25, 1.44 | 0.011 | 0.641 | -0.823, 0.369 | 0.61 | -0.14 |
| M4 - M1 | 0.631, 1.72 | 0.0008 | 0.913 | -0.0278, 0.739 | 0.11 | 0.467 |
| M5 - M1 | 0.966, 1.98 | 0.0 | 1.18 | 0.222, 0.915 | 0.0066 | 0.798 |

**Supplementary table 3.** Confident interval, p-value and cohen d estimated for the mean differences between the baseline (M1) and the rest of the moments estimated using a bootstrapping procedure of 5000 re-sampling with replacement for the time reaction in the Go/NoGo test.

|  | Intervention group | | | Control group | | |
| --- | --- | --- | --- | --- | --- | --- |
|  | 95%CI | p-value | cohen-d | 95%CI | p-value | cohen-d |
| M2 - M1 | -2.29e+02, -58.3 | 0.0016 | -0.766 | -1.1e+02, 1.37e+02 | 0.723 | 0.0986 |
| M3 - M1 | -1.84e+02, 6.69 | 0.083 | -0.435 | -65.3, 1.77e+02 | 0.376 | 0.255 |
| M4 - M1 | -2.42e+02, -41.7 | 0.0062 | -0.767 | -1.43e+02, 1.15e+02 | 0.845 | -0.0614 |
| M5 - M1 | -2.54e+02, -77.5 | 0.0006 | -0.922 | -1.71e+02, 31.1 | 0.239 | -0.343 |

**Supplementary table 4.** Confident interval, p-value and cohen d estimated for the mean differences between the baseline (M1) and the rest of the moments estimated using a bootstrapping procedure of 5000 re-sampling with replacement for the correct answers in the Visual search test.

|  | Intervention group | | | Control group | | |
| --- | --- | --- | --- | --- | --- | --- |
|  | 95%CI | p-value | cohen-d | 95%CI | p-value | cohen-d |
| M2 - M1 | -0.603, 1.05 | 0.565 | 0.127 | -0.741, 1.37] | 0.516 | 0.164 |
| M3 - M1 | -0.283, 1.24 | 0.203 | 0.295 | -1.49, 0.444 | 0.241 | -0.315 |
| M4 - M1 | -0.751, 1.14 | 0.526 | 0.153 | -1.58, 0.398 | 0.278 | -0.325 |
| M5 - M1 | -0.536, 1.21 | 0.476 | 0.183 | -1.44, 0.582 | 0.434 | -0.219 |

**Supplementary table 5.** Confident interval, p-value and cohen d estimated for the mean differences between the baseline (M1) and the rest of the moments estimated using a bootstrapping procedure of 5000 re-sampling with replacement for the the time reaction in the Visual search test.

|  | Intervention group | | | Control group | | |
| --- | --- | --- | --- | --- | --- | --- |
|  | 95%CI | p-value | cohen-d | 95%CI | p-value | cohen-d |
| M2 - M1 | -9.72e+02, -1.25e+02 | 0.0232 | -0.554 | -8.15e+02, 1.17e+02 | 0.112 | -0.441 |
| M3 - M1 | -1.01e+03, -1.27e+02 | 0.0182 | -0.58 | -9.81e+02, -1.11e+02 | 0.0292 | -0.635 |
| M4 - M1 | -1.25e+03, -4.01e+02 | 0.0028 | -0.852 | -1.17e+03, -2.94e+02 | 0.0042 | -0.887 |
| M5 - M1 | -1.48e+03, -6.46e+02 | 0.0002 | -1.14 | -1.4e+03, -6.29e+02 | 0.0 | -1.34 |

**Supplementary table 6.** Confident interval, p-value and cohen d estimated for the mean differences between the baseline (M1) and the rest of the moments estimated using a bootstrapping procedure of 5000 re-sampling with replacement for the correct answers in the Mental rotation test.

|  | Intervention group | | | Control group | | |
| --- | --- | --- | --- | --- | --- | --- |
|  | 95%CI | p-value | cohen-d | 95%CI | p-value | cohen-d |
| M2 vs M1 | -1.8, -0.0693 | 0.043 | -0.495 | -2.48, -0.926 | 0.0 | -1.09 |
| M3 - M1 | -0.267, 1.2 | 0.172 | 0.319 | -0.731, 0.894 | 0.781 | 0.0746 |
| M4 - M1 | 0.326, 1.84 | 0.0058 | 0.798 | 0.0444, 1.65 | 0.0482 | 0.601 |
| M5 - M1 | 1.16, 2.35 | 0.0 | 1.4 | 0.0529, 1.45 | 0.0538 | 0.555 |

|  |  | Wald chi-quadrat | Df | Cramer's V |
| --- | --- | --- | --- | --- |
| Correct answer GoNoGo | Group * Time | 8,392 | 4 | 0.74 |
| GoNoGo  Time | Group * Time | 11,837 | 4 | 0.88 |
| Correct answer  Visual search | Group * Time | 5,526 | 4 | 0.60 |
| Visual search  Time | Group * Time | 0,261 | 4 | 0.13 |
| Correct answer  Mental rotation | Group * Time | 5,394 | 4 | 0.59 |

**Supplementary table 7.** Calculation of the size of the effect of Cramer's equation V using the root of wald chi-quadrat divided by the sample number (n = 61) multiplied by the value of the degree of freedom (Df) found in the group and time interaction.
